# Association between HSP90 and Her2 in Gastric and Gastroesophageal Carcinomas

**DOI:** 10.1371/journal.pone.0069098

**Published:** 2013-07-11

**Authors:** Sabina Berezowska, Alexander Novotny, Karina Bauer, Annette Feuchtinger, Julia Slotta-Huspenina, Karen Becker, Rupert Langer, Axel Walch

**Affiliations:** 1 Institute of Pathology, University of Bern, Bern, Switzerland; 2 Department of Surgery, Klinikum rechts der Isar, Technische Universität München, Munich, Germany; 3 Institute of Pathology, Technische Universität München, Munich, Germany; 4 Institute of Pathology- Research Unit Analytical Pathology, Helmholtz Zentrum München, German Research Center for Environmental Health, Neuherberg, Germany; The Chinese University of Hong Kong, Hong Kong

## Abstract

**Background:**

Her2 expression and amplification occurs in a significant subset of gastro-esophageal carcinomas. Her2 is a client protein of molecular chaperones, e.g. heat shock protein (HSP) 90, rendering targeted therapies against Her2/HSP90 an interesting approach. This study aimed to investigate the role and relationship of Her2 and HSP90 in gastric and gastro-esophageal adenocarcinomas.

**Material and Methods:**

Immunohistochemical determination of HSP90 and Her2 expression was performed on 347 primary resected tumors. Her2 amplification was additionally determined by fluorescence in situ hybridization for all cases. Expression and amplification results were correlated with pathologic parameters (UICC pTNM category, tumor grading) and survival.

**Results:**

Elevated Her2 copy numbers were observed in 87 tumors, 21 of them showing amplification. 174 tumors showed Her2 immunoreactivity/expression. HSP 90 immunoreactivity was found in 125 tumors. There was no difference between gastric carcinomas and carcinomas of the gastroesophageal junction regarding Her2 or HSP90. Both high HSP90 and Her2 expression/amplification were associated with earlier tumor stages (p<0.01), absence of lymph node metastases (p<0.02) and Laurens intestinal type (p<0.001). HSP90 correlated with Her2 expression and amplification (p<0.001 each). Expressions of HSP90 and Her2, but not Her2 amplification were associated with better prognosis (p=0.02; p=0.004; p=0.802). Moreover, Her2 expression was an independent prognostic factor for overall survival in the subgroup of gastric carcinoma patients (p=0.014) besides pT category, pN category and distant metastases.

**Conclusion:**

Her2 expression and gene amplification occurred in a significant subset of cases. Our results suggest a favorable prognostic impact of Her2 expression. This warrants further investigations regarding the significance of Her2 non-amplified tumors showing Her2 immunoreactivity and the definition of Her2 status in gastric cancers. Moreover, the correlation of Her2 expression with the expression of Her2 chaperoning HSP90 may indicate a synergistic regulation. Targeting HSP90 with or without Her2 may offer additional therapeutic options for gastric carcinoma treatment.

## Introduction

Amplification and overexpression of Her2 occurs in a significant number of gastroesophageal adenocarcinomas [1–3]. Recently, Her2 targeted therapy with trastuzumab has been introduced in the treatment of metastatic gastric carcinomas and adenocarcinomas of the gastroesophageal junction [4–7]. Her2 is a client protein of HSP90, a member of the family of heat shock proteins (HSPs), which are considered molecular chaperones, as they are responsible for the correct folding of denatured or translated proteins [8,9]. It has been suggested that HSP90 expression may also modulate the effects of oncogenic Her2 [10], representing a potential mechanism of resistance to Her2 directed drugs. On the other hand, Hsp90 inhibitors may potentiate the effects of anti-cancer drugs targeting client proteins of HSP90 [11]. In breast cancer, for example, additional targeting of HSP90 has been shown to increase trastuzumab efficiency in vivo and in vitro [12,13]. Similar results have been published very recently as well for gastric carcinoma [14]. The few existing ex vivo studies about the impact of the expression and regulation of HSPs in gastric cancer show conflicting results about the prognostic role of HSP90 expression, but they describe a frequent overexpression of this potentially targetable molecule [15–17]. Any possible relationship between HSP90 and Her2, however, has not been investigated in this cancer entity to date.

The aim of the present study was to evaluate the relationship between Her2 and HSP90 in gastric carcinomas and carcinomas of the gastroesophageal junction, and its influence on tumor biology and behavior.

## Materials and Methods

### 1. Ethics statement

All patients gave written informed consent for the use of additional molecular analysis at the time of operation. The usage of human archival tissue for molecular analysis was approved by the local Ethics Committee of the Faculty of Medicine of the Technische Universität München.

### 2. Patients and tissues

We investigated formalin fixed, paraffin embedded (FFPE) archival cancer tissue from 347 patients with primary resected gastric carcinoma and carcinoma of the gastroesophageal junction who underwent surgery between 1995 and 2005 at the Klinikum Rechts der Isar of the Technische Universität München (Germany). None of the patients had received pre- or perioperative neoadjuvant treatment.

Two hundred twenty-one of the patients were male (63.7%) and 126 female (36.3%), with a median age of 69 years (range: 29 to 100). Median overall survival (OS) of all patients was 19 months (95% CI 14-23 months). Seventy-three tumors (21.0%) were adenocarcinomas of the gastroesophageal junction, and 274 were gastric carcinomas (79%). Most tumors showed an intestinal phenotype (153, 44.1%). Sixty tumors were mixed type carcinomas according to Lauren (17.3%), 111 showed a diffuse phenotype (32%) and 23 were unclassifiable (6.6%). Tumor grading was G1 (well differentiated) in 1 case (0.3%), G2 (moderately differentiated) in 54 cases (15.6%) and G3-G4 (poorly differentiated) in 292 cases (84.1%). Complete resection was achieved in 197 patients (56.8%, R0). For the purpose of this study, all tumors were reclassified according to the current UICC TNM-classification [18]. We included tumors of all TNM categories. The clinicopathologic characteristics of the collective are given in table 1. The complete dataset of the collective including the results of the immunohistochemical and in situ hybridization analysis is given as supplemental data file (Table S1).

**Table 1:** Clinicopathologic parameters.

**Characteristics**		**N**	**%**
Gender	Female	126	36.3
	Male	221	63.7

Localisation	Gastroesophageal junction	73	21.0
	Stomach	274	79.0

***pTcategory***	pT1	24	6.9
	pT2	31	8.9
	pT3	113	32.6
	pT4	179	51.6

***pNcategory***	pN0	84	24.2
	pN1	52	14.9
	pN2	51	14.7
	pN3a	118	34.0
	pN3b	42	12.1

***cMcategory***	absent	259	74.6
	present	88	25.4

***Grading***	G1-G2	55	15.9
	G3-G4	292	84.1

***Lauren´stype***	intestinal	153	44.1
	non-intestinal	194	55.9

### 3. Immunohistochemistry

Immunohistochemistry was performed on FFPE tissue. Preparation of tissue microarrays (TMA) was performed as described before, generating triplicate cores from randomly selected tumor areas with a diameter of 1.0 mm each [19]. The paraffin blocks were freshly cut (3 µm). Slides were dewaxed and rehydrated, with subsequent heat-induced antigen retrieval using 10 mM citrate buffer, pH 6, H_2_O_2_ blocking using 3% H_2_O_2_ in aqua destillata and avidin biotin blocking (Avidin/Biotin blocking kit, Vector Laboratories, Inc., Burlingame, CA, USA). The sections were then incubated with antibodies for HSP90 (Abcam, Cambridge, UK) and Her2 (DAKO, Glostrup, DK). Positive and negative controls were included in each reaction.

**Figure 1:**
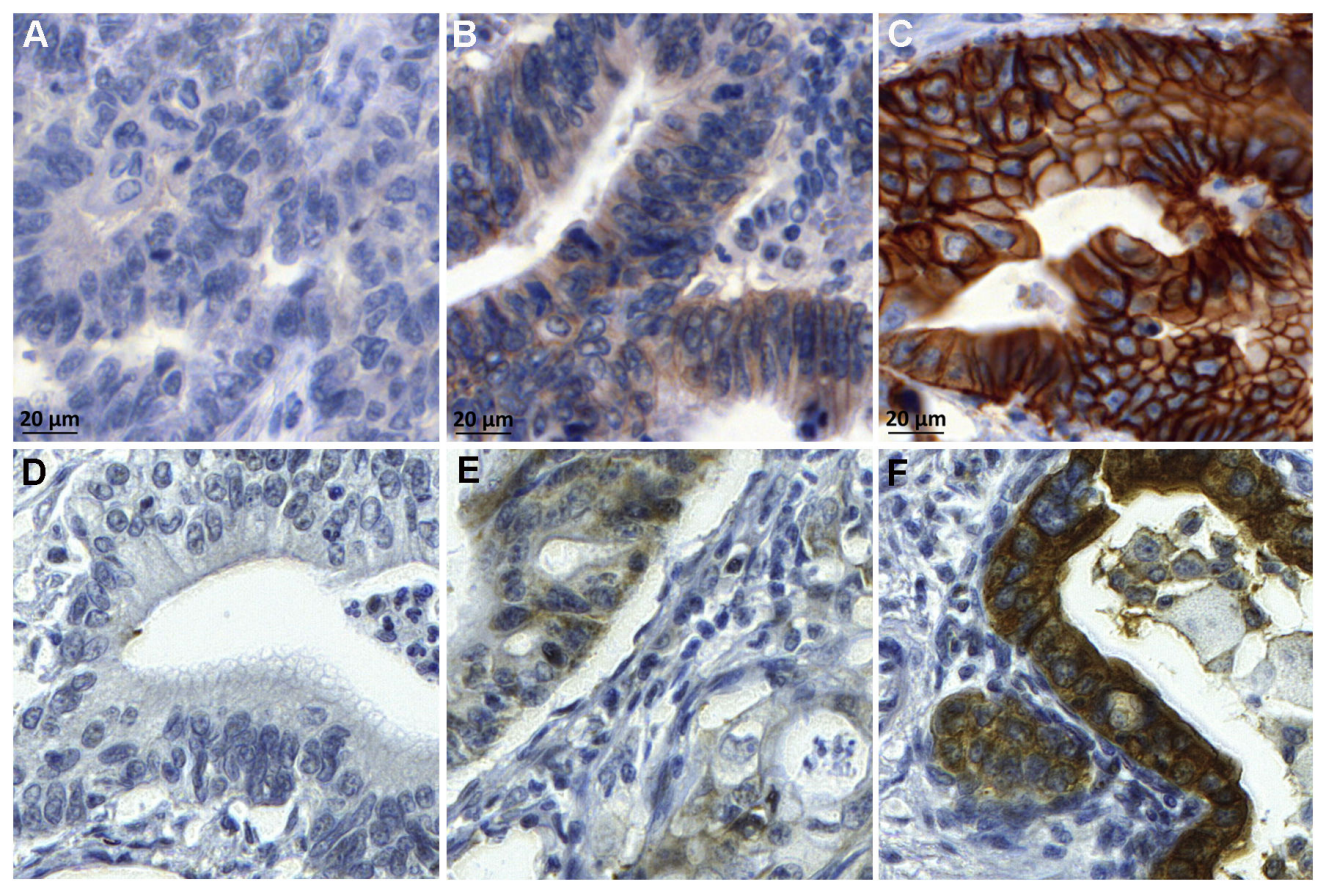
Her2 and HSP90 expression in gastric adenocarcinoma. Gastric adenocarcinoma with (A) negative, (B) score 1+, (C) score 3+ Her2 expression; (D) negative HSP90, (E) low HSP90 and (F) high HSP90 expression.

Positive HSP90 staining was defined as cytoplasmic staining of ≥10% of carcinoma cells (Figure 1). Her2 expression on TMA cores was assessed according to published recommendations for routine Her2 evaluation in gastric carcinoma, including the slight modifications recommended for the use on biopsies [20]: In short, immunohistochemistry 3+ staining was defined as any membranous staining visible at low magnification (objective × 2.5–5), immunohistochemistry 2+ was defined as membranous staining visible at × 10–20 magnification, and immunohistochemistry 1+ staining was defined as weak membranous staining visible only with × 40 magnification. Cases with no visible membranous reactivity were classified as negative (Figure 1).

Evaluation of HSP90 and Her2 expression was performed by two independent observers (SB, RL or AW) and discrepancies were discussed at a multihead microscope to gain a final consent. Only cores with technically unequivocal staining results and sufficient tumor content (>50 tumor cells) were used for final analysis.

### 4. Fluorescence in situ Hybridization

**Figure 2:**
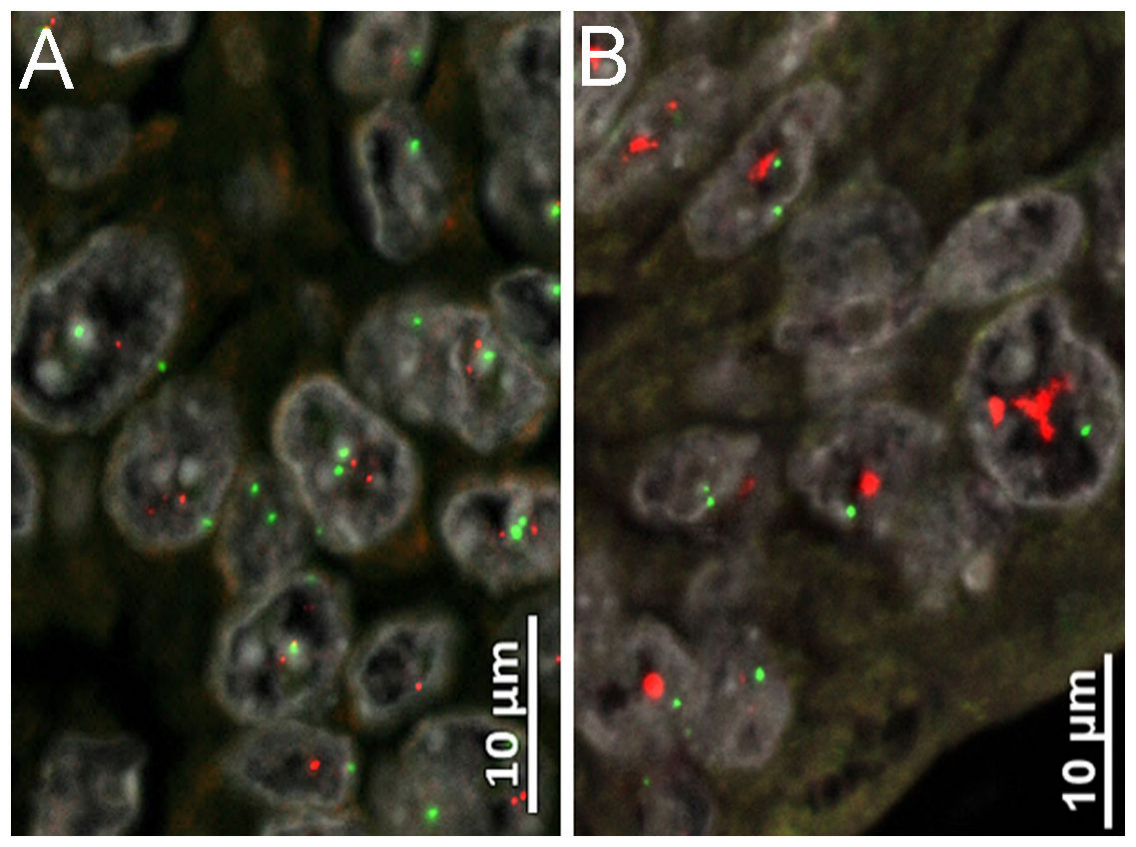
Fluorescence in situ analysis of Her2. (A) Disomy and (B) high level Her2 amplification.

All cases were also tested for Her2 amplification by fluorescence in situ hybridization (FISH), irrespective of prior immunohistochemical Her2 results. An assay with fluorescence-labeled locus-specific DNA probes for Her2 and chromosome-17 centromeric α-satellite (Chrombios) was hybridized onto 4 μm TMA sections as described before [21,22]. FISH signal evaluation was performed by visual counting using an epifluorescence microscope (Zeiss Axioplan 2, Carl Zeiss Microimaging GmbH) according to standard procedures as recommended in literature [21]. At least 50 invasive tumor cells per case with a minimum of one signal for Her2 gene and centromere(CEP)-17 were randomly selected, and the mean Her2 and CEP17 count was calculated. Cases were classified as amplified when Her2/CEP17 quotient was ≥2. Cases with simultaneously elevated Her2 and CEP17 counts were assigned as polysome when the Her2/CEP17 quotient was <2 [21] (Figure 2).

### 5. Statistical Analysis

For statistical analysis, IBM SPSS 21.0 Statistics statistical software (SPSS Inc., Chicago, IL, USA) was used. Associations between immunohistochemical expression patterns, results of FISH analysis and pathological features were given in crosstabs and were evaluated with X^²^ and Fisher’s exact test. Survival analysis was performed using Kaplan-Meier estimates, log rank tests and Cox’s proportional hazards regression analysis. All tests were 2-sided, and the significance level was set at 0.05.

## Results

### 1. Her2 expression and amplification

Three hundred thirty-six cases were evaluable for membranous Her2 expression and all 347 cases for Her2 amplification. The majority of tumors showed Her2 expression (174 cases; 51.8%), which was weak in 96 cases (1+; 28.6%), moderate in 43 (2+; 12.4%), and strong in 35 tumor samples (3+; 10.1%; Figure 1 A–C Table 2). Eleven cases could not be evaluated by immunohistochemistry using the inclusion criteria given above.

**Table 2:** Comparison between Her2 immunohistochemistry and FISH (p<0.001).

		Her2IHC				Total
		0	1+	2+	3+	
Her2FISH	Negative (n=260)	131	72	31	17	251
	Polysomy (n=66)	31	17	11	5	64
	Amplification (n=21)	0	7	1	13	21
Total		162	96	43	35	336

Fluorescence in situ hybridization analysis showed elevated Her2 copy numbers in 87 tumors, 66 of them were polysome (19%) and 21 patients (6.1%) showed Her2 amplification as defined by a Her2/CEP17 ratio ≥2 (Figure 2 Table 2). Two hundred sixty cases (74.9%) showed no Her2 amplification.

Correlation was strong between Her2 expression and amplification (p<0.001, Table 2). All Her2 amplified cases showed membranous Her2 expression, which was strong (3+) in 61,9%, and contrasted the predominantly weak Her2 expression in polysome tumors. None of the immunohistochemically Her2-negative (score 0) tumors were Her2 amplified; however, 31 tumors had elevated Her2 copy numbers (polysomy). Moreover, a significant number of Her2 expressing tumors (3+; 22/35; 63%) failed to show Her2 amplification (17 disome, 5 polysome). Additionally, 24 cases with weak immunostaining (score 1+) had elevated Her2 copy numbers, with seven of them showing Her2 amplification (Table 2).

Applying the current FDA and EMEA algorithm, which defines Her2 positivity as either immunohistochemical score 3+ or score 2+ validated by Her2 amplification assessment, 300 patients (89.3%) would have been considered Her2 negative, and 36 (10.7%) as Her2 positive [20].

### 2. HSP90 expression

Of the 323 cases evaluable for HSP90 expression, immunoreactivity was found in 125 tumors (38.7%). Only 6 cases (1.8%) showed a strong reaction against HSP90 versus a weak cytoplasmic staining in the other positive cases (Figure 1 D–F). In 24 cases no valid immunohistochemical analysis for HSP90 was possible, due to technical reasons.

### 3. Association between Her2 and HSP90

There was no difference between gastric carcinomas and carcinomas of the gastroesophageal junction regarding Her2 or HSP90. HSP90 expression correlated with Her2 expression and Her2-status according to FDA and EMEA (see chapter 3.1.; p<0.001 each; Table 3), but not with Her2 amplification alone (p=0.067).

**Table 3:** Association between HSP90 expression and Her2 expression and Her2 status according to FDA/EMEA (p<0.001 each).

		**Her2 expression**			**Her2 status**	
		negative	positive (*)		negative	positive (**)
**HSP90** (n=323)	Negative (n=198)	112	81		182	11
	Positive (n=125)	39	85		100	24
**Total**		151	166		282	35

*score 1+,2+,3+; **according to the FDA/EMEA

### 4. Clinicopathological parameters and survival analysis

HSP90, Her2 expression and Her2 status according to FDA and EMEA were associated with lower local tumor burden, absence of lymph node metastases, better tumor differentiation (grading), and intestinal phenotype according to Lauren (p values see Tables 4 and 5). No such associations could be demonstrated evaluating Her2 amplification alone.

**Table 4:** HSP90 expression and pathological parameters.

**factor**		**HSP90**		
		***neg***	***pos***	***p-value***
***pTcategory***	pT1	4	19	p<0.001
	pT2	16	14	
	pT3	59	45	
	pT4	119	47	
				
***pNcategory***	pN0	41	39	p=0.024
	pN1	24	24	
	pN2	32	16	
	pN3	101	46	
				
***cMcategory***	absent	138	103	p=0.012
	present	60	22	
				
***Grading***	G1-G2	25	29	p=0.025
	G3-G4	173	96	
				
***Lauren***	intestinal	65	84	p<0.001
	non-int.	131	40	

**Table 5:** Her2 expression and Her2 status and pathological parameters.

**factor**		**Her2 expression**				**Her2 status**		
		***neg***	pos(*)	***p-value***		***neg***	pos(**)	***p-value***
pT category	pT1	5	19	p=0.005		16	8	p=0.002
	pT2	11	20			27	4	
	pT3	51	58			100	9	
	pT4	95	77			157	15	

pN category	pN0	30	51	p=0.108 (p=0.014)#		67	14	p=0.128 (p=0.038)#
	pN1	24	27			47	4	
	pN2	22	27			42	7	
	pN3	86	69			144	11	

cM category	absent	115	138	p=0.1		224	29	p=0.542
	present	47	36			76	7	

Grading	G1-G2	14	41	p<0.001		37	18	p<0.001
	G3-G4	148	133			263	18	

Lauren	intestinal	48	101	p<0.001		120	29	p<0.001
	non-int.	112	72			178	6	

* Immunohistochemical score 1+,2+,3+; **according to the FDA and EMEA; #for pNneg vs. pNpos

**Figure 3:**
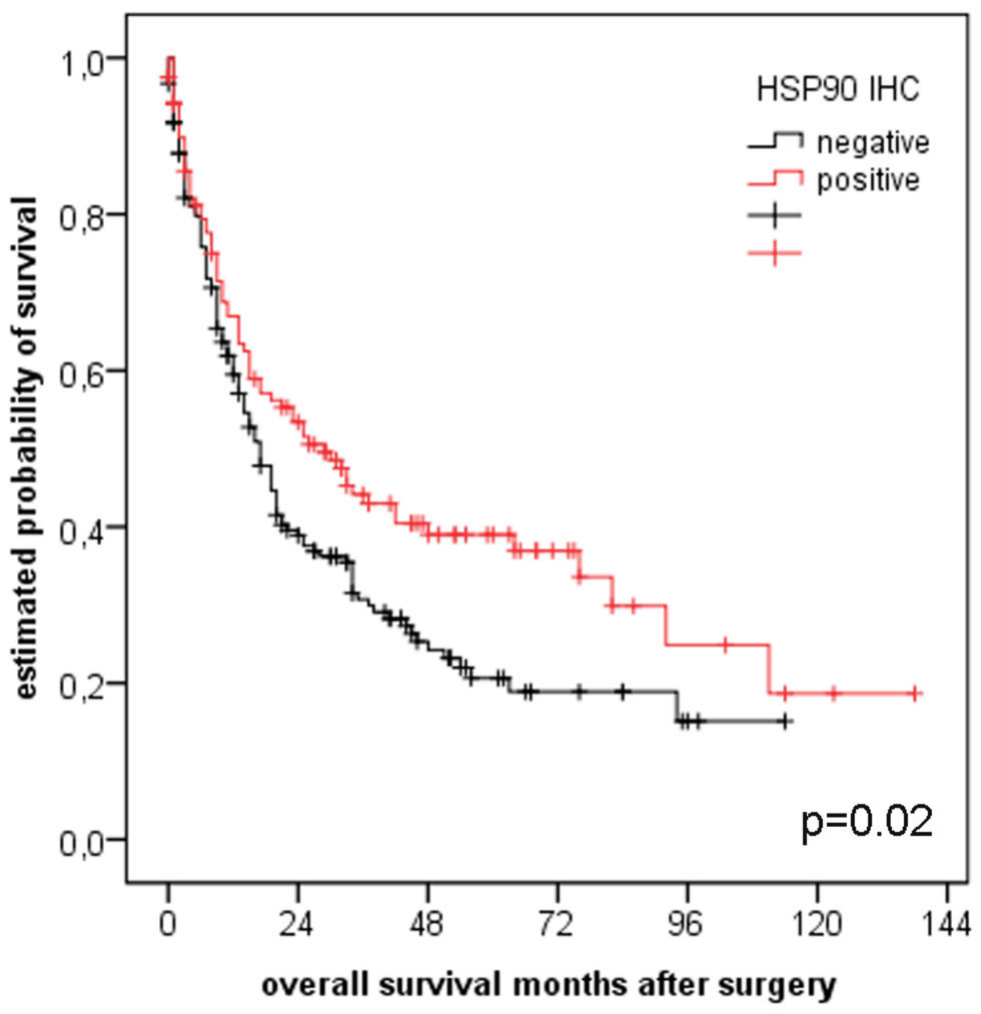
Survival analysis for HSP90. Univariate analysis showed a strong association between HSP90 expression and better prognosis (n=323, p=0.02).

**Figure 4:**
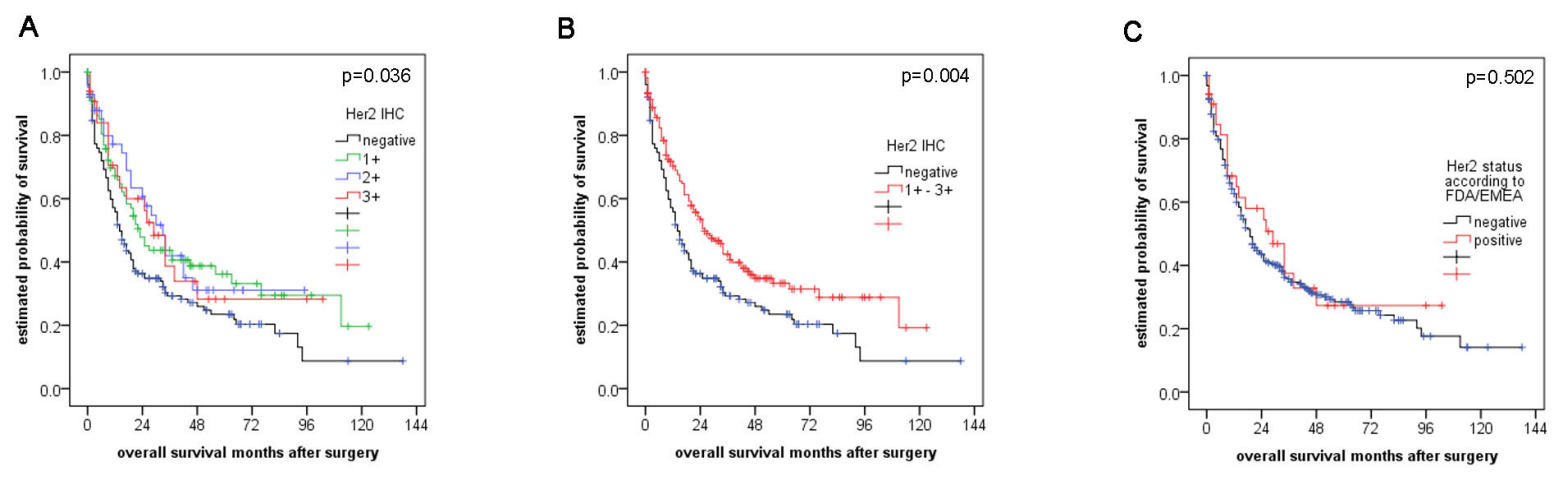
Survival analysis for Her2 expression and Her2 status according to FDA/EMEA. (A, B) Her2 expression, but not (C) Her2 status according to FDA/EMEA was associated with better prognosis in univariate analysis.

Expression of HSP90 (p=0.02) and Her2 (score 1+, 2+ and 3+; p=0.004), but not Her2 status according to the FDA/EMEA (p=0.502), Her2 amplification alone (p=0.802) or elevated Her2 copy numbers (p=0.813) were associated with better prognosis in univariate analysis (Figures 3 and 4). Additional prognostic factors were UICC pT category (p<0.001), presence of lymph node or distant metastases at the time of surgery (p<0.001 each), resection status (p<0.001), younger age at the time of the operation (p=0.024) and intestinal phenotype according to Lauren (p<0.001). Grading (tumor differentiation) or localization (proximal versus distal) had no impact on overall survival. However, HSP90 or Her2 were not independent prognostic factors in multivariate analysis in the whole collective. When analyzing the large group of gastric cancer patients separately though (n=274), presence of any Her2 immunoreactivity (score 1+, 2+ and 3+) emerged as an independent prognostic factor for overall survival (p=0.014) besides pT category, pN category and distant metastases (Table 6). The independent prognostic role of Her2 expression (p=0.024), pT, pN and distant metastases was retained in the subgroup of non-intestinal type tumors (Table 7). Contrary, Her2 expression was not an independent prognostic factor in to the subgroup of purely intestinal type tumors.

**Table 6:** Multivariate Analysis for the subgroup of gastric cancer patients (n=274).

**factor**	**Exp(B)**	**95.0% CI for Exp(B)**		**p-value**
		min	max	
**pTcategory**	1.753	1.335	2.302	<0.001
**pNcategory**	1.283	1.108	1.487	0.001
**cMcategory**	1.556	1.041	2.327	0.031
**Grading**	0.715	0.447	1.144	0.162
**Lauren**	0.952	0.662	1.370	0.790
**Resection-status**	1.193	0.897	1.586	0.226
Her2 expression*	0.658	0.472	0.917	0.014

* Immunohistochemical score 1+,2+,3+

**Table 7:** Multivariate Analysis for the subgroup of non-intestinal gastric and gastroesophageal carcinomas (n=194).

**factor**	**Exp(B)**	**95.0% CI for Exp(B)**		**p-value**
		min	max	
**pTcategory**	1.983	1.361	2.888	<0.001
**pNcategory**	1.36	1.156	1.599	<0.001
**cMcategory**	1.581	1.032	2.422	0.035
**Grading**	0.638	0.278	1.462	0.288
**Resection-status**	0.962	0.68	1.36	0.825
Her2 expression*	0.643	0.437	0.944	0.024

* Immunohistochemical score 1+,2+,3+

## Discussion

Gastric carcinomas and adenocarcinomas of the gastroesophageal junction have been shown to express Her2 in a significant number of cases, rendering it a possible valuable molecule for molecular targeting [1–4,7,20]. However, there is a high diversity of the definition of Her2 status in gastric cancer in literature. The definition depends on the detection methods that are used (immunohistochemistry; in situ hybridization) and on the interpretation of the results of the staining and hybridization. For immunohistochemistry, where the proposals of Hofmann and Ruschoff [20,23] are widely accepted as evaluation standard, usually a score 3+ is considered as overexpression. A score of 2+ is considered as equivocal, and a score of 1+ and 0 is considered as negative. However, there are some studies, which consider also weak immunostaining (1+) as a positive reaction [3]. In our study we based the description of Her2 expression (1+, 2+ and 3+) on the results of the survival analysis, where the prognostic impact of a weak immunoreactivity (i.e. 1+) was identical to a 2+ and 3+ immunoscore. Additional and corresponding FISH analysis, which would characterize the amplification status at a genomic level, is only performed in a subset of studies [2,3,7]. A strength of our study is that we present corresponding immunohistochemistry and FISH results of a large number of primary resected gastric carcinomas and carcinomas of the gastroesophageal junction. More than half of the tumors in our case collection of 347 specimens expressed varying degrees of Her2. Ninety-six of them showed only weak immunoreactivity. These cases would have been initially regarded “Her2 negative” by organ specific established scoring systems, without further evaluation by FISH, following the recommendations of the FDA/EMEA: Both institutions recommend that only tumors with an immunoscore of 3+ and tumors with 2+ and additional confirming in situ hybridization should be labeled as Her2 positive and may be candidates for trastuzumab therapy [20,24]. However, as discussed later, our results indicate a certain biologic significance of even weak Her2 immunoreactivity, and argue in favor of considering every positive staining as an indicator for increased Her2 expression. Her2 gene copy number showed a strong correlation with Her2 expression and none of the Her2 negative tumors (immunohistochemically score 0) was amplified. This is in line with observations reported by others [2,3]. Like expected, Her2 expression or amplification was significantly more frequently observed in tumors of intestinal type and better differentiation [25]. Interestingly, a quarter of cases with a weak immunoreactivity (score 1+) showed elevated Her2 copy numbers and a subset of those displayed Her2-amplification. These cases would have been missed following the FDA/EMEA algorithm. Similar results have been reported recently, recommending that not only Her2 immunohistochemically 2+, but also 1+ gastric carcinomas should be evaluated by FISH analysis [26]. We observed a considerable number of discordant cases with either Her2 amplification without significant Her2 expression or vice versa. One explanation for those differences in single cases might be intratumoral heterogeneity of Her2, which has been reported to occur in a significant percentage of gastric cancers [27,28]. We used the same cores within our TMAs to assess both Her2 amplification and expression, so that we can reliably exclude that different tumor areas were chosen for immunohistochemical and FISH analysis, and rather consider our observation as a tumorbiological true finding. The usage of TMAs for biomarker analysis has been shown to be a powerful tool for analyzing molecular markers in large tissue collections with the advantage of homogenous reaction conditions, thus avoiding false negative or false positive staining or hybridization results due to technical reasons. However, some limitations have been reported for molecular alterations with heterogeneous expression patterns [29]. We used a TMA that was constructed for the analysis of various biomarkers in gastric cancer [19]. The cores were randomly taken from various areas of the tumors. The number of three cores is considered as appropriate as a “rule of thumb” covering certain amounts of heterogeneity, avoiding significant missing of information due to loss of cores and allowing the inclusion of even smaller tumors where no more TMA cores could be taken [29]. For assessing Her2 in breast cancer, even less than three cores have been reported to yield satisfactory results [30,31]. For gastric cancer, there are several reports about the limitation of assessing Her2 on superficially taken gastric cancer biopsies due to intratumoral heterogeneity [28,32,33]. These studies have pointed out the risk of false negative results due to missing overexpressed clones. Most studies, though, regarded an immunoscore of 1+ as negative [28,34]. Moreover, intratumoral heterogeneity was reported to be more pronounced for immunohistochemical detection than gene amplification [33,34]. In our study we report any immunoreactivity and have corresponding FISH data for every tumor. The TMA cores were randomly selected covering central and peripheral areas, and not only superficial areas of the tumor. Moreover, the rate of Her2 “positive” cases lies within the range reported in literature. At last, we chose the proposed modification by Ruschoff et al. for assessing Her2 on biopsies [20] for the immunohistochemical evaluation of Her2 on three TMA cores for each tumor, as we did in our previous study on esophageal adenocarcinomas, which show similar degrees of intratumoral heterogeneity of Her2 [21,35,36]. We therefore consider our approach as appropriate for the purpose of this explorative study, yet being aware of its potential weakness and accepting a certain rate of both Her2 and HSP90 false negative tumors. However, the relatively high number of cases and the advantages of homogenous technical conditions may equilibrate this limitation. In case of clinical management, the use of TMAs may harbor the same risk of missing information with consecutive incorrect therapeutic decisions, and in that context investigation of whole tissue sections should strongly be favored over the use of smaller samples [20].

One interesting finding of our study was the considerable rate of cases which were classified as polysome, i.e. expressing elevated Her2 and CEP17 copy numbers below a Her2/CEP17 quotient >2, which is the recommended definition of Her2 amplification. Most of these cases were Her2 1+ or 2+. In breast cancer, there are several publications, which direct towards this yet unclear issue of Her2/CEP17 polysomy in terms of determination of true Her2 status, and there is increasing evidence that Her2/CEP17 polysomy represents rather a phenomenon of co-amplification than true polysomy [37–39].

Studies in breast cancer have also pointed out the limitations of assessment of Her2 status by immunohistochemistry and additional FISH. Immunohistochemistry has been described to lack objectivity producing false-positive or -negative outcomes due to interobserver variability, and both immunohistochemistry and FISH are heavily dependent from technical issues such as fixation and buffering [40,41]. However, major efforts with respect to standardization of protocols and evaluation systems have improved the rate of discordance between immunohistochemistry and FISH results over the last years [42–44]. In consequence, the estimated rate of incorrectly assessed Her2 could be lowered to less than 5% [45]. The evaluation system by the group around Ruschoff, which was applied in the present work, takes into account the tumor specific characteristics of Her2 staining in gastric cancer as opposed to breast carcinoma, and represents a first step towards standardization of Her2 assessment in this tumor entity [20,23].

In literature there are congruent data about the rate of Her2 overexpressed and amplified tumors in gastric cancer but there are still inconsistent results regarding any prognostic value of Her2 in gastric carcinomas and adenocarcinomas of the gastroesophageal junction [2,3,7]. A relatively high number of papers advocating a negative impact of high Her2 levels on survival are faced by a considerable amount of reports which could demonstrate no or the opposite association of Her2 and prognosis [5,46–48]. Using the definition of Her2 positivity according to the FDA/EMEA criteria, which are widely applied for assessing Her2 in gastric cancer, we would not have been able to demonstrate any significant impact on prognosis. This would also have been the case if we had considered an immunoscore of 2+ and 3+ as criterion for Her2 expression, like it was done in other immunohistochemical studies [48]. In contrast, we observed that in our case collection any Her2 immunoreactivity – which also comprised a weak staining (1+) – was associated with less aggressive tumor behavior and turned out to be an independent significant favorable prognostic marker both in the group of gastric carcinoma patients and in the subgroup of non-intestinal type tumors, which showed predominantly weak Her2 immunoreactivity. Thus our results go in line with the few reports that demonstrate a favorable prognostic impact of higher tumoral Her2 expression, e.g. in esophageal adenocarcinomas [49].

Our observation of the prognostic impact of even weak Her2 immunoreactivity was unexpected, especially with regard to the reports of others [3], but represents a highly reliable finding. The large case collection it is based on comprises all stages of primary resected tumors without pre- or perioperative chemotherapy, originates from a single center in Germany and can be regarded as representative in terms of gender and age distribution of the patients, and the prognostic impact of established parameters such as stage and grade. Moreover, the strength of the present study is that every tumor was analyzed for both Her2 expression and amplification, which is comparably provided in recent studies only.

Our results speak in favor of a questioning attitude towards the assessment of Her2 in gastric and gastroesophageal carcinomas, like it has been adopted for Her2 in breast cancer for almost two decades now [50].

Given the likelihood of increased application of trastuzumab or other Her2 directed agents in gastric and gastroesophageal cancer and the increasing number of publications about Her2 in these tumors there will be clearly a need for an exact definition of “Her2 status” that will cover yet unclear findings, like polysomy, heterogeneity [27,28] and cases which lack correlation between gene amplification and expression. This definition should also cover predictive and potentially prognostic value in order to provide a robust tool for further therapeutic decisions in the treatment of gastric cancer patients.

The stability and maturation of Her2 has been shown to be mediated by so called “molecular chaperones” belonging to the family of heat shock proteins (HSPs) [8,9]. HSPs are highly conserved proteins, which are responsible for the accurate folding of other proteins, thereby maintaining cellular integrity and homeostasis [51]. There is evidence, that deregulation of HSPs can be observed in malignant diseases – which may be due to intrinsic antiapoptotic effects but also the altered interaction with other oncogenic molecules [52,53]. One of the most abundant cellular HSPs is HSP90. HSP90 interacts with a large number of proteins, amongst them tyrosine kinases such as Her2 and EGFR, where the interaction with the cytoplasmic kinase domain leads to protein stabilization, but also signaling proteins like Akt, K-ras, Raf-1, and mutated signaling proteins like p53 and v-Src [54,55]. Therefore, HSP90 represents a unique player in cellular homeostasis, and, in consequence, is also regarded as a potential antitumoral target, especially in Her2 positive tumors [55,56].

Inhibitors of HSP90 including Geldanamycin and its derivates (e.g., 17-AAG and 17-DMAG) have already entered clinical application [57–59]. In gastric cancer, preclinical studies of HSP90 inhibitors alone and in combination with other chemotherapeutic drugs or trastuzumab have already been performed [10,60,61]. There are already promising data about enhancing trastuzumab efficacy or even overcoming trastuzumab resistance through HSP90 targeting for breast and also gastric cancer in vitro and in vivo [12–14,62,63]. In addition, dual Her2/HSP90 targeting drugs are being developed [64].

We could verify the postulated association between Her2 and HSP90 expression on the tissue level, and could demonstrate the prognostic role of Her2/HSP90. This points towards a co-regulation of both molecules in vivo. Furthermore, elevated HSP90 levels may render tumors susceptible for anti-HSP90 directed therapy, a prerequisite met by one third of cases of our case collective. Considering recent pharmaceutical advances, either combination therapy with conventional drugs could be a possible approach, or - with regard to the high association with Her2 expression – also and especially as additional approach to anti-Her2 therapy [65]. According to in vitro data [13,66], HSP90 inhibition should result in enhancing the effect of therapy directed against Her2 or could even represent a possible tool for overcoming Her2 resistance. In human gastric cancer, various expression levels of HSP90 can be detected, which may serve as a tissue-based rationale for targeting this molecule. The impact of HSP90 expression on prognosis in gastric cancer patients, however, remains currently unclear: there have been conflicting data about an adverse or - like in our study – favorable influence of HSP90 in gastric carcinomas in different populations and collectives [17].

In summary, we could demonstrate immunoreactivity for Her2 and corresponding gene amplification in a significant subset of gastric and gastroesophageal adenocarcinomas. Our results suggest a favorable prognostic impact of Her2 expression. This warrants further investigations regarding the significance of Her2 non-amplified tumors showing Her2 immunoreactivity on the one hand and the definition of Her2 status in gastric cancers on the other hand. Moreover, the correlation with the expression of Her2 chaperoning HSP90 may indicate a synergistic regulation of these molecules. Targeting HSP90 with or without Her2 may offer additional therapeutic options for gastric carcinoma treatment.

## Supporting Information

Table S1 Complete patient dataset. (XLS)

## References

[1] Hechtman JF, Polydorides AD (2012) HER2/neu gene amplification and protein overexpression in gastric and gastroesophageal junction adenocarcinoma: a review of histopathology, diagnostic testing, and clinical implications. Arch Pathol Lab Med 136: 691-697. doi:10.5858/arpa.2011-0168-RS. PubMed: 22646280. 22646280 10.5858/arpa.2011-0168-RS

[2] Jørgensen JT, Hersom M (2012) HER2 as a Prognostic Marker in Gastric Cancer - A Systematic Analysis of Data from the Literature. J Cancer 3: 137-144. doi:10.7150/jca.4090. PubMed: 22481979. 22481979 10.7150/jca.4090 PMC3319979

[3] Chua TC, Merrett ND (2012) Clinicopathologic factors associated with HER2-positive gastric cancer and its impact on survival outcomes--a systematic review. Int J Cancer 130: 2845-2856. doi:10.1002/ijc.26292. PubMed: 21780108. 21780108 10.1002/ijc.26292

[4] Bang YJ, Van Cutsem E, Feyereislova A, Chung HC, Shen L et al. (2010) Trastuzumab in combination with chemotherapy versus chemotherapy alone for treatment of HER2-positive advanced gastric or gastro-oesophageal junction cancer (ToGA): a phase 3, open-label, randomised controlled trial. Lancet 376: 687-697. doi:10.1016/S0140-6736(10)61121-X. PubMed: 20728210. 20728210 10.1016/S0140-6736(10)61121-X

[5] Shitara K, Yatabe Y, Matsuo K, Sugano M, Kondo C et al. (2013) Prognosis of patients with advanced gastric cancer by HER2 status and trastuzumab treatment. Gastric Cancer 16: 261-267. doi:10.1007/s10120-012-0179-9. PubMed: 22797858. 22797858 10.1007/s10120-012-0179-9

[6] Grávalos C, Gómez-Martín C, Rivera F, Alés I, Queralt B et al. (2011) Phase II study of trastuzumab and cisplatin as first-line therapy in patients with HER2-positive advanced gastric or gastroesophageal junction cancer. Clin Transl Oncol 13: 179-184. doi:10.1007/s12094-011-0637-6. PubMed: 21421462. 21421462 10.1007/s12094-011-0637-6

[7] Maresch J, Schoppmann SF, Thallinger CM, Zielinski CC, Hejna M (2012) Her-2/neu gene amplification and over-expression in stomach and esophageal adenocarcinoma: from pathology to treatment. Crit Rev Oncol/Hematol 82: 310-322. doi:10.1016/j.critrevonc.2011.06.003. PubMed: 21783379. 10.1016/j.critrevonc.2011.06.003 21783379

[8] Sidera K, Gaitanou M, Stellas D, Matsas R, Patsavoudi E (2008) A critical role for HSP90 in cancer cell invasion involves interaction with the extracellular domain of HER-2. J Biol Chem 283: 2031-2041. PubMed: 18056992. 18056992 10.1074/jbc.M701803200

[9] Citri A, Harari D, Shohat G, Ramakrishnan P, Gan J et al. (2006) Hsp90 recognizes a common surface on client kinases. J Biol Chem 281: 14361-14369. doi:10.1074/jbc.M512613200. PubMed: 16551624. 16551624 10.1074/jbc.M512613200

[10] Lang SA, Klein D, Moser C, Gaumann A, Glockzin G et al. (2007) Inhibition of heat shock protein 90 impairs epidermal growth factor-mediated signaling in gastric cancer cells and reduces tumor growth and vascularization in vivo. Mol Cancer Ther 6: 1123-1132. doi:10.1158/1535-7163.MCT-06-0628. PubMed: 17363505. 17363505 10.1158/1535-7163.MCT-06-0628

[11] Neckers L, Workman P (2012) Hsp90 molecular chaperone inhibitors: are we there yet? Clin Cancer Res 18: 64-76. doi:10.1158/1078-0432.MECHRES-B64. PubMed: 22215907. 22215907 10.1158/1078-0432.CCR-11-1000 PMC3252205

[12] Modi S, Stopeck A, Linden H, Solit D, Chandarlapaty S et al. (2011) HSP90 inhibition is effective in breast cancer: a phase II trial of tanespimycin (17-AAG) plus trastuzumab in patients with HER2-positive metastatic breast cancer progressing on trastuzumab. Clin Cancer Res 17: 5132-5139. doi:10.1158/1078-0432.CCR-11-0072. PubMed: 21558407. 21558407 10.1158/1078-0432.CCR-11-0072

[13] Scaltriti M, Serra V, Normant E, Guzman M, Rodriguez O et al. (2011) Antitumor activity of the Hsp90 inhibitor IPI-504 in HER2-positive trastuzumab-resistant breast cancer. Mol Cancer Ther 10: 817-824. doi:10.1158/1535-7163.MCT-10-0966. PubMed: 21383049. 21383049 10.1158/1535-7163.MCT-10-0966

[14] Lu C, Liu D, Jin J, Deokar H, Zhang Y et al. (2013) Inhibition of gastric tumor growth by a novel Hsp90 inhibitor. Biochem Pharmacol 85: 1246-1256. doi:10.1016/j.bcp.2013.02.003. PubMed: 23415900. 23415900 10.1016/j.bcp.2013.02.003 PMC3617064

[15] Giaginis C, Daskalopoulou SS, Vgenopoulou S, Sfiniadakis I, Kouraklis G et al. (2009) Heat Shock Protein-27, -60 and -90 expression in gastric cancer: association with clinicopathological variables and patient survival. BMC Gastroenterol 9: 14. doi:10.1186/1471-230X-9-14. PubMed: 19203381. 19203381 10.1186/1471-230X-9-14 PMC2644705

[16] Buffart TE, Carvalho B, van Grieken NC, van Wieringen WN, Tijssen M et al. (2012) Losses of chromosome 5q and 14q are associated with favorable clinical outcome of patients with gastric cancer. Oncologist 17: 653-662. doi:10.1634/theoncologist.2010-0379. PubMed: 22531355. 22531355 10.1634/theoncologist.2010-0379 PMC3360905

[17] Wang J, Cui S, Zhang X, Wu Y, Tang H (2013) High expression of heat shock protein 90 is associated with tumor aggressiveness and poor prognosis in patients with advanced gastric cancer. PLOS ONE 8: e62876. doi:10.1371/journal.pone.0062876. PubMed: 23638161. 23638161 10.1371/journal.pone.0062876 PMC3637377

[18] Sobin L, Gospodarowicz ML, Wittekind C (2010) TNM classification of malignant tumors; New York: John Wiley & Sons.

[19] Balluff B, Elsner M, Kowarsch A, Rauser S, Meding S et al. (2010) Classification of HER2/neu status in gastric cancer using a breast-cancer derived proteome classifier. J Proteome Res 9: 6317-6322. doi:10.1021/pr100573s. PubMed: 21058730. 21058730 10.1021/pr100573s

[20] Rüschoff J, Hanna W, Bilous M, Hofmann M, Osamura RY et al. (2012) HER2 testing in gastric cancer: a practical approach. Mod Pathol 25: 637-650. doi:10.1038/modpathol.2011.198. PubMed: 22222640. 22222640 10.1038/modpathol.2011.198

[21] Rauser S, Weis R, Braselmann H, Feith M, Stein HJ et al. (2007) Significance of HER2 low-level copy gain in Barrett’s cancer: implications for fluorescence in situ hybridization testing in tissues. Clin Cancer Res 13: 5115-5123. doi:10.1158/1078-0432.CCR-07-0465. PubMed: 17785566. 17785566 10.1158/1078-0432.CCR-07-0465

[22] Walch A, Specht K, Braselmann H, Stein H, Siewert JR et al. (2004) Coamplification and coexpression of GRB7 and ERBB2 is found in high grade intraepithelial neoplasia and in invasive Barrett’s carcinoma. Int J Cancer 112: 747-753. doi:10.1002/ijc.20411. PubMed: 15386389. 15386389 10.1002/ijc.20411

[23] Hofmann M, Stoss O, Shi D, Büttner R, van de Vijver M et al. (2008) Assessment of a HER2 scoring system for gastric cancer: results from a validation study. Histopathology 52: 797-805. doi:10.1111/j.1365-2559.2008.03028.x. PubMed: 18422971. 18422971 10.1111/j.1365-2559.2008.03028.x

[24] EMEA (2009) Committee for medicinal products for human use post-authorisation summary of positive opinion for herceptin. Available: www.emea.europa.eu/pdfs/human/opinion/Herceptin_82246709en.pdf. Accessed 2013 March 1.

[25] Tanner M, Hollmén M, Junttila TT, Kapanen AI, Tommola S et al. (2005) Amplification of HER-2 in gastric carcinoma: association with Topoisomerase IIalpha gene amplification, intestinal type, poor prognosis and sensitivity to trastuzumab. Ann Oncol 16: 273-278. doi:10.1093/annonc/mdi064. PubMed: 15668283. 15668283 10.1093/annonc/mdi064

[26] Grillo F, Fassan M, Ceccaroli C, Giacometti C, Curto M et al. (2013) The Reliability of Endoscopic Biopsies in Assessing HER2 Status in Gastric and Gastroesophageal Junction Cancer: A Study Comparing Biopsies with Surgical Samples. Transl Oncol 6: 10-16. PubMed: 23418612. 23418612 10.1593/tlo.12334 PMC3573649

[27] Lee HE, Park KU, Yoo SB, Nam SK, Park do J et al. (2013) Clinical significance of intratumoral HER2 heterogeneity in gastric cancer. Eur J Cancer 49: 1448-1457. doi:10.1016/j.ejca.2012.10.018. PubMed: 23146959. 23146959 10.1016/j.ejca.2012.10.018

[28] Warneke VS, Behrens HM, Böger C, Becker T, Lordick F et al. (2013) Her2/neu testing in gastric cancer: evaluating the risk of sampling errors. Ann Oncol 24: 725-733. doi:10.1093/annonc/mds528. PubMed: 23139264. 23139264 10.1093/annonc/mds528 PMC3574551

[29] Ilyas M, Grabsch H, Ellis IO, Womack C, Brown R et al. (2013) Guidelines and considerations for conducting experiments using tissue microarrays. Histopathology 62: 827-839. doi:10.1111/his.12118. PubMed: 23672312. 23672312 10.1111/his.12118

[30] Thomson TA, Zhou C, Chu C, Knight B (2009) Tissue microarray for routine analysis of breast biomarkers in the clinical laboratory. Am J Clin Pathol 132: 899-905. doi:10.1309/AJCPW37QGECDYCDO. PubMed: 19926582. 19926582 10.1309/AJCPW37QGECDYCDO

[31] Zhang D, Salto-Tellez M, Do E, Putti TC, Koay ES (2003) Evaluation of HER-2/neu oncogene status in breast tumors on tissue microarrays. Hum Pathol 34: 362-368. doi:10.1053/hupa.2003.60. PubMed: 12733117. 12733117 10.1053/hupa.2003.60

[32] Pirrelli M, Caruso ML, Di Maggio M, Armentano R, Valentini AM (2013) Are biopsy specimens predictive of HER2 status in gastric cancer patients? Dig Dis Sci 58: 397-404. PubMed: 22918687. 22918687 10.1007/s10620-012-2357-3

[33] Yang J, Luo H, Li Y, Li J, Cai Z et al. (2012) Intratumoral heterogeneity determines discordant results of diagnostic tests for human epidermal growth factor receptor (HER) 2 in gastric cancer specimens. Cell Biochem Biophys 62: 221-228. doi:10.1007/s12013-011-9286-1. PubMed: 21927816. 21927816 10.1007/s12013-011-9286-1

[34] Yoon Cho E, Park K, Do I, Cho J, Kim J et al. (2013) Heterogeneity of ERBB2 in gastric carcinomas: a study of tissue microarray and matched primary and metastatic carcinomas. Mod Pathol 26: 677-684. doi:10.1038/modpathol.2012.205. PubMed: 23238628. 23238628 10.1038/modpathol.2012.205

[35] Walch A, Specht K, Bink K, Zitzelsberger H, Braselmann H et al. (2001) Her-2/neu gene amplification, elevated mRNA expression, and protein overexpression in the metaplasia-dysplasia-adenocarcinoma sequence of Barrett’s esophagus. Lab Invest 81: 791-801. doi:10.1038/labinvest.3780289. PubMed: 11406641. 11406641 10.1038/labinvest.3780289

[36] Langer R, Rauser S, Feith M, Nährig JM, Feuchtinger A et al. (2011) Assessment of ErbB2 (Her2) in oesophageal adenocarcinomas: summary of a revised immunohistochemical evaluation system, bright field double in situ hybridisation and fluorescence in situ hybridisation. Mod Pathol 24: 908-916. doi:10.1038/modpathol.2011.52. PubMed: 21516080. 21516080 10.1038/modpathol.2011.52

[37] Tse CH, Hwang HC, Goldstein LC, Kandalaft PL, Wiley JC et al. (2011) Determining true HER2 gene status in breast cancers with polysomy by using alternative chromosome 17 reference genes: implications for anti-HER2 targeted therapy. J Clin Oncol 29: 4168-4174. doi:10.1200/JCO.2011.36.0107. PubMed: 21947821. 21947821 10.1200/JCO.2011.36.0107

[38] Vranic S, Teruya B, Repertinger S, Ulmer P, Hagenkord J et al. (2011) Assessment of HER2 gene status in breast carcinomas with polysomy of chromosome 17. Cancer 117: 48-53. doi:10.1002/cncr.25580. PubMed: 20803611. 20803611 10.1002/cncr.25580

[39] Marchiò C, Lambros MB, Gugliotta P, Di Cantogno LV, Botta C et al. (2009) Does chromosome 17 centromere copy number predict polysomy in breast cancer? A fluorescence in situ hybridization and microarray-based CGH analysis. J Pathol 219: 16-24. doi:10.1002/path.2574. PubMed: 19670217. 19670217 10.1002/path.2574

[40] Vani K, Sompuram SR, Fitzgibbons P, Bogen SA (2008) National HER2 proficiency test results using standardized quantitative controls: characterization of laboratory failures. Arch Pathol Lab Med 132: 211-216. PubMed: 18251579. 18251579 10.5858/2008-132-211-NHPTRU

[41] Hicks DG, Tubbs RR (2005) Assessment of the HER2 status in breast cancer by fluorescence in situ hybridization: a technical review with interpretive guidelines. Hum Pathol 36: 250-261. doi:10.1016/j.humpath.2004.11.010. PubMed: 15791569. 15791569 10.1016/j.humpath.2004.11.010

[42] Dowsett M, Hanna WM, Kockx M, Penault-Llorca F, Rüschoff J et al. (2007) Standardization of HER2 testing: results of an international proficiency-testing ring study. Mod Pathol 20: 584-591. doi:10.1038/modpathol.3800774. PubMed: 17396141. 17396141 10.1038/modpathol.3800774

[43] Wolff AC, Hammond ME, Schwartz JN, Hagerty KL, Allred DC et al. (2007) American Society of Clinical Oncology/College of American Pathologists guideline recommendations for human epidermal growth factor receptor 2 testing in breast cancer. J Clin Oncol 25: 118-145. PubMed: 17159189. 17159189 10.1200/JCO.2006.09.2775

[44] Sauter G, Lee J, Bartlett JM, Slamon DJ, Press MF (2009) Guidelines for human epidermal growth factor receptor 2 testing: biologic and methodologic considerations. J Clin Oncol 27: 1323-1333. doi:10.1200/JCO.2007.14.8197. PubMed: 19204209. 19204209 10.1200/JCO.2007.14.8197

[45] Dekker TJ, Borg ST, Hooijer GK, Meijer SL, Wesseling J et al. (2012) Determining sensitivity and specificity of HER2 testing in breast cancer using a tissue micro-array approach. Breast Cancer Res 14: R93. doi:10.1186/bcr3208. PubMed: 22694844. 22694844 10.1186/bcr3208 PMC3446356

[46] Terashima M, Kitada K, Ochiai A, Ichikawa W, Kurahashi I et al. (2012) Impact of expression of human epidermal growth factor receptors EGFR and ERBB2 on survival in stage II/III gastric cancer. Clin Cancer Res 18: 5992-6000. doi:10.1158/1078-0432.CCR-12-1318. PubMed: 22977193. 22977193 10.1158/1078-0432.CCR-12-1318

[47] Janjigian YY, Werner D, Pauligk C, Steinmetz K, Kelsen DP et al. (2012) Prognosis of metastatic gastric and gastroesophageal junction cancer by HER2 status: a European and USA International collaborative analysis. Ann Oncol 23: 2656-2662. doi:10.1093/annonc/mds104. PubMed: 22689179. 22689179 10.1093/annonc/mds104

[48] Grabsch H, Sivakumar S, Gray S, Gabbert HE, Müller W (2010) HER2 expression in gastric cancer: Rare, heterogeneous and of no prognostic value - conclusions from 924 cases of two independent series. Cell Oncol 32: 57-65. PubMed: 20208134. 20208134 10.3233/CLO-2009-0497 PMC4619246

[49] Yoon HH, Shi Q, Sukov WR, Wiktor AE, Khan M et al. (2012) Association of HER2/ErbB2 expression and gene amplification with pathologic features and prognosis in esophageal adenocarcinomas. Clin Cancer Res 18: 546-554. doi:10.1158/1078-0432.CCR-11-2272. PubMed: 22252257. 22252257 10.1158/1078-0432.CCR-11-2272 PMC3261584

[50] Hicks DG, Kulkarni S (2008) HER2+ breast cancer: review of biologic relevance and optimal use of diagnostic tools. Am J Clin Pathol 129: 263-273. doi:10.1309/99AE032R9FM8WND1. PubMed: 18208807. 18208807 10.1309/99AE032R9FM8WND1

[51] Lindquist S, Craig EA (1988) The heat-shock proteins. Annu Rev Genet 22: 631-677. doi:10.1146/annurev.ge.22.120188.003215. PubMed: 2853609. 2853609 10.1146/annurev.ge.22.120188.003215

[52] Calderwood SK, Khaleque MA, Sawyer DB, Ciocca DR (2006) Heat shock proteins in cancer: chaperones of tumorigenesis. Trends Biochem Sci 31: 164-172. doi:10.1016/j.tibs.2006.01.006. PubMed: 16483782. 16483782 10.1016/j.tibs.2006.01.006

[53] Ciocca DR, Calderwood SK (2005) Heat shock proteins in cancer: diagnostic, prognostic, predictive, and treatment implications. Cell Stress Chaperones 10: 86-103. doi:10.1379/CSC-99r.1. PubMed: 16038406. 16038406 10.1379/CSC-99r.1 PMC1176476

[54] Porter JR, Fritz CC, Depew KM (2010) Discovery and development of Hsp90 inhibitors: a promising pathway for cancer therapy. Curr Opin Chem Biol 14: 412-420. doi:10.1016/j.cbpa.2010.03.019. PubMed: 20409745. 20409745 10.1016/j.cbpa.2010.03.019

[55] Hong DS, Banerji U, Tavana B, George GC, Aaron J et al. (2013) Targeting the molecular chaperone heat shock protein 90 (HSP90): lessons learned and future directions. Cancer Treat Rev 39: 375-387. doi:10.1016/j.ctrv.2012.10.001. PubMed: 23199899. 23199899 10.1016/j.ctrv.2012.10.001

[56] Scaltriti M, Dawood S, Cortes J (2012) Molecular pathways: targeting hsp90--who benefits and who does not. Clin Cancer Res 18: 4508-4513. doi:10.1158/1078-0432.CCR-11-2138. PubMed: 22718860. 22718860 10.1158/1078-0432.CCR-11-2138

[57] Lu X, Xiao L, Wang L, Ruden DM (2012) Hsp90 inhibitors and drug resistance in cancer: the potential benefits of combination therapies of Hsp90 inhibitors and other anti-cancer drugs. Biochem Pharmacol 83: 995-1004. doi:10.1016/j.bcp.2011.11.011. PubMed: 22120678. 22120678 10.1016/j.bcp.2011.11.011 PMC3299878

[58] Patel HJ, Modi S, Chiosis G, Taldone T (2011) Advances in the discovery and development of heat-shock protein 90 inhibitors for cancer treatment. Expert Opin Drugs Discov 6: 559-587. doi:10.1517/17460441.2011.563296. PubMed: 22400044. 10.1517/17460441.2011.563296 PMC3293194 22400044

[59] Jego G, Hazoumé A, Seigneuric R, Garrido C (2013) Targeting heat shock proteins in cancer. Cancer Lett 332: 275-285. doi:10.1016/j.canlet.2010.10.014. PubMed: 21078542. 21078542 10.1016/j.canlet.2010.10.014

[60] Lee KH, Lee JH, Han SW, Im SA, Kim TY et al. (2011) Antitumor activity of NVP-AUY922, a novel heat shock protein 90 inhibitor, in human gastric cancer cells is mediated through proteasomal degradation of client proteins. Cancer Sci 102: 1388-1395. doi:10.1111/j.1349-7006.2011.01944.x. PubMed: 21453385. 21453385 10.1111/j.1349-7006.2011.01944.x

[61] Ono N, Yamazaki T, Nakanishi Y, Fujii T, Sakata K et al. (2012) Preclinical antitumor activity of the novel heat shock protein 90 inhibitor CH5164840 against human epidermal growth factor receptor 2 (HER2)-overexpressing cancers. Cancer Sci 103: 342-349. doi:10.1111/j.1349-7006.2011.02144.x. PubMed: 22050138. 22050138 10.1111/j.1349-7006.2011.02144.x

[62] Chandarlapaty S, Scaltriti M, Angelini P, Ye Q, Guzman M et al. (2010) Inhibitors of HSP90 block p95-HER2 signaling in Trastuzumab-resistant tumors and suppress their growth. Oncogene 29: 325-334. doi:10.1038/onc.2009.337. PubMed: 19855434. 19855434 10.1038/onc.2009.337 PMC3057066

[63] Wainberg ZA, Anghel A, Rogers AM, Desai AJ, Kalous O et al. (2013) Inhibition of HSP90 with AUY922 Induces Synergy in HER2-Amplified Trastuzumab-Resistant Breast and Gastric Cancer. Mol Cancer Ther 12: 509-519. doi:10.1158/1535-7163.MCT-12-0507. PubMed: 23395886. 23395886 10.1158/1535-7163.MCT-12-0507

[64] Chen CY, Chen CY (2010) Insights into designing the dual-targeted HER2/HSP90 inhibitors. J Mol Graph Modell 29: 21-31. doi:10.1016/j.jmgm.2010.04.002. PubMed: 20471294. 10.1016/j.jmgm.2010.04.002 20471294

[65] Citri A, Kochupurakkal BS, Yarden Y (2004) The achilles heel of ErbB-2/HER2: regulation by the Hsp90 chaperone machine and potential for pharmacological intervention. Cell Cycle 3: 51-60. PubMed: 14657666. 14657666

[66] Jhaveri K, Taldone T, Modi S, Chiosis G (2012) Advances in the clinical development of heat shock protein 90 (Hsp90) inhibitors in cancers. Biochim Biophys Acta 1823: 742-755. doi:10.1016/j.bbamcr.2011.10.008. PubMed: 22062686. 22062686 10.1016/j.bbamcr.2011.10.008 PMC3288123

